# Sexual and reproductive health services utilization by female sex workers is context-specific: results from a cross-sectional survey in India, Kenya, Mozambique and South Africa

**DOI:** 10.1186/s12978-017-0277-6

**Published:** 2017-01-19

**Authors:** Yves Lafort, Ross Greener, Anuradha Roy, Letitia Greener, Wilkister Ombidi, Faustino Lessitala, Jolene Skordis-Worrall, Mags Beksinska, Peter Gichangi, Sushena Reza-Paul, Jenni A. Smit, Matthew Chersich, Wim Delva

**Affiliations:** 1 International Centre for Reproductive Health, Ghent University, Ghent, Belgium; 2 MatCH Research Unit (Maternal, Adolescent and Child Health Research Unit), Faculty of Health Sciences, University of the Witwatersrand, Durban, South Africa; 3 Ashodaya Samithi, Mysore, India; 4 International Centre for Reproductive Health-Kenya, Mombasa, Kenya; 5 International Centre for Reproductive Health-Mozambique, Maputo, Mozambique; 6 Institute for Global Health, University College London, London, UK; 7 University of Nairobi, Nairobi, Kenya; 8 University of Manitoba, Winnipeg, Canada; 9 Wits Reproductive Health and HIV Institute, Faculty of Health Sciences, University of the Witwatersrand, Johannesburg, South Africa; 10 The South African DST-NRF Centre of Excellence in Epidemiological Modelling and Analysis (SACEMA), University of Stellenbosch, Stellenbosch, South Africa; 11 Center for Statistics, Hasselt University, Diepenbeek, Belgium

**Keywords:** Female sex workers, Sexual and reproductive health, Care seeking behaviour, Sub-Saharan Africa, India

## Abstract

**Background:**

Female sex workers (FSWs) are extremely vulnerable to adverse sexual and reproductive health (SRH) outcomes. To mitigate these risks, they require access to services covering not only HIV prevention but also contraception, cervical cancer screening and sexual violence. To develop context-specific intervention packages to improve uptake, we identified gaps in service utilization in four different cities.

**Methods:**

A cross-sectional survey was conducted, as part of the baseline assessment of an implementation research project. FWSs were recruited in Durban, South Africa (*n* = 400), Mombasa, Kenya (*n* = 400), Mysore, India (*n* = 458) and Tete, Mozambique (*n* = 308), using respondent-driven sampling (RDS) and starting with 8-16 ‘seeds’ identified by the peer educators. FSWs responded to a standardised interviewer-administered questionnaire about the use of contraceptive methods and services for cervical cancer screening, sexual violence and unwanted pregnancies. RDS-adjusted proportions and surrounding 95% confidence intervals were estimated by non-parametric bootstrapping, and compared across cities using post-hoc pairwise comparison tests with Dunn–Šidák correction.

**Results:**

Current use of any modern contraception ranged from 86.2% in Tete to 98.4% in Mombasa (*p* = 0.001), while non-barrier contraception (hormonal, IUD or sterilisation) varied from 33.4% in Durban to 85.1% in Mysore (*p* < 0.001). Ever having used emergency contraception ranged from 2.4% in Mysore to 38.1% in Mombasa (*p* < 0.001), ever having been screened for cervical cancer from 0.0% in Tete to 29.0% in Durban (*p* < 0.001), and having gone to a health facility for a termination of an unwanted pregnancy from 15.0% in Durban to 93.7% in Mysore (*p* < 0.001). Having sought medical care after forced sex varied from 34.4% in Mombasa to 51.9% in Mysore (*p* = 0.860). Many of the differences between cities remained statistically significant after adjusting for variations in FSWs’ sociodemographic characteristics.

**Conclusion:**

The use of SRH commodities and services by FSWs is often low and is highly context-specific. Reasons for variation across cities need to be further explored. The differences are unlikely caused by differences in socio-demographic characteristics and more probably stem from differences in the availability and accessibility of SRH services. Intervention packages to improve use of contraceptives and SRH services should be tailored to the particular gaps in each city.

## Plain english summary

Sex workers are a severely stigmatised population and extremely vulnerable to adverse health outcomes related to having frequent sexual intercourse, such as unwanted pregnancies, cervical cancer, or sexual violence. We interviewed, in four different cities in resource-limited countries, Durban in South Africa, Tete in Mozambique, Mombasa in Kenya, and Mysore in India, a representative sample of female sex workers and explored to what extent they are sufficiently using the services providing prevention and care for these risks. The extent of use differed greatly between cities. In Durban only one in three (33%) of the interviewed sex workers was using an effective contraceptive method, while in Mysore this was 85%. The morning-after pill was commonly used in Mombasa (38% had ever used it), but not in Mysore where only 2% ever used it. In Tete none had ever been screened for cervical cancer and in Durban 29% had. Seeking medical care after a sexual assault was low in all cities, ranging from 34% in Mombasa to 52% in Mysore. The sex workers substantially differed across cities in age, educational level, mobility, marital status and number of clients, and we assessed if this was the reason for the differences in service use, but it was not. We suspect that the main reason is a difference in the availability of services and in barriers to access. Interventions to improve the use of services have therefore to be tailored to the particular gaps in each setting.

## Background

Female sex workers (FSWs) are amongst the most vulnerable for adverse sexual and reproductive health (SRH) outcomes, because of multiple sexual contacts with different partners [1]. In low-resource countries, their vulnerability is further exacerbated by poverty, endemic violence, criminalisation and repeated human rights violations [2], low community cohesion, and heavy episodic drinking [1]. High levels of mobility and illegal immigration status, lack of familiarity with locally available health services, fear of stigmatisation and discrimination at public services, and unsuitable opening hours constitute important barriers to healthcare for these women [3, 4].

Programmes with FSWs in sub-Saharan Africa and South Asia have generally focussed on HIV prevention and care and rarely address access to other SRH services, such as contraception, care for unwanted pregnancies, cervical cancer screening, and sexual and gender-based violence (SGBV) services [5, 6]. Several studies have shown that the use of effective contraception is often insufficient and unwanted pregnancies are common [7–9]. Female sex workers often use condoms as the sole contraceptive method, which has high failure rates among sex workers [7–11]. Human papilloma virus infection, and resulting cervical cancer, are frequent [12, 13], but little is known about the extent to which screening programmes are reaching FSWs. In addition, FSWs are often marginalised and are therefore commonly victims of harassment and violence [2, 14], but the extent to which support services for SGBV are utilised by this population has rarely been documented. In the context of an implementation research project to improve SRH among FSWs, we therefore assessed gaps in the use of SRH services, other than HIV prevention and care, in four settings in Africa and India, and evaluated to what extent use of SRH services was context-specific.

## Methods

### Contextual background

The study was conducted in three cities in Africa and one in India where sex work is common, criminalised and FSWs are marginalised. Durban, South Africa, has a population of 3.4 million and an estimated FSW population of 6300 [15]. The Tete-Moatize area in Mozambique has approximately 250,000 inhabitants and an enumeration exercise counted 4415 FSWs in 2008 [16]. Mombasa, Kenya, with a population of about 1.2 million has approximately 9000 FSWs [17]. Mysore, in Karnataka State India, has approximately 900,000 inhabitants and about 2000 FSWs. Female sex worker-targeted services are provided by non-governmental organisations in each site. Services in Durban are delivered primarily through outreach, while in the three other cities service provision occurs within stand-alone clinics. In Tete-Moatize this consists of a small clinic on the outskirts of Moatize [16], in Mombasa there are three drop-in clinics in different divisions of the city [18] and in Mysore there is a clinic operated by the FSWs organisation Ashodaya Samithi [19].

The Diagonal Interventions to Fast-Forward Enhanced Reproductive Health (DIFFER) study is a 5-year implementation research project that aims at improving access to HIV/SRH services for FSWs by a better linkage between interventions targeted at FSWs and the general health services [20]. It applies a methodological framework for health systems research, starting with a detailed situation and policy analysis, followed by the development of context-specific packages of interventions to improve access to SRH services in each city. These packages will combine a strengthening of services specifically targeted at FSWs with improving access to the general health services, and developing linkage systems between both. The performance of the packages is evaluation after at least 18 months of implementation [21]. The baseline situational analysis used a mixed-methods approach combining quantitative surveys among FSWs and SRH service clients, with qualitative focus group discussions and key informant interviews. To assess the effect of the intervention, quantifiable indicators of SRH commodities and services utilization are measured at baseline and end-line through a cross-sectional survey among a representative sample of FSWs in each city. This paper reports on the baseline cross-sectional survey, presenting a comparison of the indicators of use of contraception and services for cervical cancer screening, unwanted pregnancies and sexual violence, by FSWs across the four cities. The findings on the use of HIV prevention and care services and of where FSWs seek care have been published elsewhere [22, 23]. The findings on the policy analysis and the other - qualitative - components of the situational analysis are also available [24–26].

### Cross-sectional surveys

We recruited FSWs using Respondent-Driven-Sampling (RDS), which is similar to snowballing, but corrects for the bias towards FSWs with large social networks through statistical adjustments [27]. Respondent-driven sampling begins with the selection of “seeds” i.e. known members of the population of interest. Each seed is asked to identify a number of other FSWs from their social network for the survey, who subsequently identify other FSWs, and so on, until the desired sample size is reached.

In Durban, Tete, Mombasa and Mysore, 11, 13, 16 and 8 seeds were recruited, respectively. Seeds were identified by experienced peer educators who work within long-standing peer outreach programmes in each city. To ensure branching into different FSW networks, seeds were categorised according to locally relevant sub-populations. In Durban, they were categorised according to age, location of soliciting (indoors/outdoors) and migration status; in Tete, according to nationality (Mozambican/Zimbabwean), place of residence (Tete city/Moatize city) and type of FSW (full-time/occasional); and in Mombasa, according to location of soliciting sex (bar/club based, street/truck based, brothel/home based, and beach based). In Durban, Tete and Mombasa each participant recruited, using coupons, up to 3 new participants and in Mysore up to 5. Handling of coupons was monitored with Electronic RDS Coupon Manager Version 3.0 in Durban, Tete and Mombasa, and manually through a coupon log notebook in Mysore.

To detect substantial changes in project indicators between the baseline survey and the end-of-project survey with a significance level of 0.05 and a power of 0.80, the minimum estimated sample size was 400 per study site. In Tete, the refusal rate was high, in particular among FSWs of Mozambican nationality, allegedly because false rumours had spread about the intentions of the survey, and recruitment was stopped at 308 FSWs because of time constraints.

Potential respondents were screened for eligibility and invited to answer a standardised, interviewer-administered questionnaire. The questionnaire collected information on socio-demographic and sex work characteristics, main contraceptive method currently used, ever having used emergency contraception, unwanted pregnancies in the last 5 years and action taken for an unwanted pregnancy, ever having tested for cervical cancer, having experienced forced sex in the past 12 months and if medical care was sought subsequently. Respondents were considered eligible to participate in the study if they were females 18 years or older and a practicing FSW (having received money or gifts for sex at least three times in the last 6 months). Eligible and consenting respondents were interviewed using either a paper-based questionnaire (Durban, Mombasa and Mysore) or Computer-Assisted Personal Interview (QDS™) software (Tete). Confidentiality was safeguarded through the use of non‐identifying survey codes and keeping all collected information locked or protected.

In Durban, Mombasa and Mysore, the questionnaires were entered in an MS-Access database and in Tete uploaded in a QDS data warehouse. The survey data were merged with the coupon data, and imported into STATA version 12. In the first stage of the analysis, we calculated population point estimates with their 95% confidence intervals (CI) by non-parametric bootstrapping, adjusted for social network size and homophily within networks, using the STATA RDS analysis package and the Volz-Heckathorn estimator [28]. Then, for the analysis comparing outcomes between cities, we performed post-hoc pairwise comparison tests after fitting a logistic regression model with RDS-adjusted weights, using jack-knife resampling and Dunn–Šidák correction for multiple comparisons [29]. The analysis focused on the comparison of the proportion using contraceptives, action taken for unwanted pregnancies, cervical cancer screening and SGBV services across the four cities. Sociodemographic sex worker characteristics that were associated with both the outcome and the city were stepwise introduced in the regression model and retained in the model if they changed the odds ratio (OR) by at least 10%. To assess overall use of SRH commodities and services we established a composite index. In the denominator we added ‘1’ for each service the FSW was in need of: a non-barrier contraceptive method if she did not want to become pregnant; ever having been screened for cervical cancer if she was older than 30 years; and seeking medical care if she had been forced to sex. In the numerator we added ‘1’ for each of these services that the FSW had used. The index thus represents the proportion of needed services that a FSW used.

The applicable ethical review boards in each country (the University of Witwatersrand’s Human Research Ethics Committee in South Africa, the National Committee of Bioethics for Health in Mozambique, the KNH/UoN Ethics and Research Committee in Kenya, and the Asha Kirana Institutional Ethics Committee in India) and the ethical board of the coordinating agency in Belgium (Commission for Medical Ethics of the University Hospital Ghent) approved the study protocols.

## Results

### Socio-demographic characteristics (Table 1)

**Table 1:** Socio-demographic and sex work characteristics

Characteristic	Durban (*N* = 400)		Tete (*N* = 308)		Mombasa (*N* = 400)		Mysore (*N* = 458)	
	RDS-Adjusted		RDS-Adjusted		RDS-Adjusted		RDS-Adjusted	
	%	95% CI	%	95% CI	%	95% CI	%	95% CI
Age (years)								
Median	27		29		26		34	
< =20	6.4	3.6 – 9.7	15.6	9.0 – 23.8	11.6	7.5 – 16.3	0.3	0.2 – 0.8
21-25	37.3	30.1 – 44.4	20.6	15.3 – 26.6	30.6	24.6 – 37.5	16.6	11.2 – 23.4
26-30	31.3	24.9 – 38.1	27.1	20.3 – 34.5	29.0	23.5 – 34.7	33.0	20.8 – 42.1
31-35	12.8	8.7 – 17.3	19.8	14.6 – 25.6	15.7	11.0 – 21.1	19.5	13.7 – 25.2
> =36	12.2	6.7 – 18.4	16.9	11.2 – 22.2	13.0	9.3 – 17.2	30.7	23.2 – 39.2
Nationality								
National	99.0	97.9 – 99.9	32.5	23.9 – 40.1	97.3	95.6 – 98.9	100.0	-
Foreign	1.0	0.1 – 2.1	67.5	59.9 – 76.1	2.7	1.1 – 4.4	0.0	-
Education								
None or less than primary	10.5	6.3 – 15.0	10.2	5.7 – 15.2	47.6	40.8 – 54.2	79.0	67.4 – 87.7
Primary completed, secondary not completed	68.7	61.4 – 75.7	69.3	62.3 – 76.0	41.1	34.8 – 47.3	16.7	8.1 – 27.8
Secondary completed or higher education	20.8	14.9 – 26.8	20.4	15.3 – 25.8	11.3	7.2 – 16.5	4.3	2.3 – 7.0
Years living in current residence								
< 3 years	39.8	32.4 – 47.4	55.0	47.4 – 62.0	56.6	49.9 – 63.2	11.6	7.0 – 17.5
> = 3 years	60.2	52.6 – 67.6	45.0	38.0 – 52.6	43.4	36.8 – 50.1	88.4	82.5 – 93.0
Was away from residence								
In the past year	56.5	48.8 – 63.3	27.4	21.6 – 33.8	48.2	41.5 – 55.1	8.5	5.1 – 13.2
Present relationship								
Married or cohabiting	28.7	22.2 – 35.4	8.2	2.9 – 15.1	1.2	0.3 – 2.3	54.1	44.0 – 6.3
Single, never married or cohabited	70.5	63.6 – 77.1	31.0	24.1 – 37.5	61.8	55.1 – 67.7	3.5	1.2 – 6.8
Single, previously married or cohabited	0.8	0.2 – 1.6	60.8	52.9 – 68.8	37.1	31.1 – 43.7	42.4	33.4 – 52.6
No of commercial sex acts in the past month								
Median	29		30		20		20	
< =15	30.6	23.3 – 37.9	15.0	10.6 – 20.2	8.8	5.7 – 12.2	41.9	31.8 – 51.7
16-25	25.0	18.8 – 31.4	26.0	18.3 – 33.0	73.3	67.6 – 78.4	55.6	45.8 – 65.5
26-40	20.9	15.2 – 27.1	32.2	24.5 – 40.6	17.6	13.1 – 22.4	2.5	0.8 – 4.6
> 40	23.5	18.0 – 29.2	26.7	20.2 – 33.2	0.3	0.2 – 0.8		
Non-commercial sex partners in the past month								
Regular partner^a^	46.8	39.6 – 54.2	33.8	26.0 – 41.0	51.7	44.9-58.3	96.8	94.2 – 98.8
Occasional partner^a^	20.2	14.7 – 25.9	48.7	40.9 – 56.5	24.0	17.7 – 30.7	59.6	50.0 – 69.4
Has other source of income								
Yes	10.5	6.5 – 15.0	19.2	13.9 – 25.1	42.6	36.3 – 49.0	27.8	21.2 – 35.1

^a^A ‘regular’ partner was defined as ‘a long-standing non-commercial partner who did not give you money or gifts in return for sex and towards whom you feel an emotional attachment’ and an occasional partner as ‘those partners other than your regular partner(s) who did not give you money or gifts in return for sex’

FSWs were on average older in Mysore than in the African cities. Educational levels were higher in the African cities than in Mysore. More than half of FSWs in Durban and Mombasa reported never have being married or cohabiting with a partner, while in Mysore this proportion was very small (3.5%). The proportion currently living with a partner, be it married or not, was particularly small in Mombasa (1.2%). In Tete most FSWs had previously been in a steady relationship, but were currently single (62%).

The number of commercial sex acts reported in the past month was substantially lower in Mysore than in the African cities. The proportion of FSWs that reported a regular non-commercial partner was much higher in Mysore (97%) and substantially more FSWs reported other non-commercial partners in Mysore and Tete (60% and 49%, respectively) than in Durban and Mombasa (20% and 24%, respectively). Fewer FSWs had another source of income in Durban (11%) than in the other cities. The most common sources of income were activities such as hairdressing, selling at the market, or waitressing, across cities.

### Occurrence of SRH risks and use of SRH commodities and services (Table 2 and 3)

**Table 2:** Occurrence of SRH risks and use of SRH commodities and services

	RDS-Adjusted %			
	Durban	Tete	Mombasa	Mysore
Currently using contraception^b^	*N = 381*	*N = 244*	*N = 388*	*N = 381*
Yes	91.3	86.2	98.4	95.8
Main contraception method used^c^	*N = 346*	*N = 212*	*371*	*373*
Injectable contraceptives	29.7	42.8	25.9	0.0
Oral contraceptives	3.2	33.3	6.4	0.6
IUD	0.1	2.4	0.8	1.0
Implant	0.0	3.5	33.0	0.3
Condom	63.7	17.0	33.3	10.0
Female sterilization	3.2	0.9	0.6	88.2
Currently using a non-barrier modern contraceptive method^b^	*N = 378*	*N = 244*	*N = 379*	*N = 379*
Yes	33.4	70.6	65.6	85.1
Ever used emergency contraception	*N = 389*	*N = 298*	*N = 400*	*N = 455*
Yes	27.9	13.4	38.1	2.4
Unwanted pregnancy in the last five years	*N = 394*	*N = 301*	*N = 345*	*N = 458*
Yes	37.6	7.5	30.6	8.0
Action taken for unwanted pregnancy^d^	*N = 150*	*N = 19*	*N = 122*	*N = 43*
Went to a health facility for an abortion	15.0	(35.9)^a^	21.9	(93.7)^a^
Kept the pregnancy	81.2	(64.1)^a^	70.8	(6.3)^a^
Went elsewhere for an abortion	3.8	(0.0) ^a^	7.3	(0.0)^a^
Ever tested for cervical cancer	*N = 400*	*N = 304*	*N = 399*	*N = 458*
Yes	29.0	0.0	14.4	11.5
Ever tested for cervical cancer^e^	*N = 124*	*N = 144*	*N = 122*	*N = 337*
Yes	43.8	0.0	21.1	13.6
Forced sex in the past 12 months	*N = 393*	*N = 264*	*N = 399*	*N = 458*
Yes	36.3	13.5	14.9	7.1
Condom use at last forced sex incident^f^	*N = 129*	*N = 38*	*N = 63*	*N = 39*
Yes	47.5	(20.4)^a^	26.2	(38.5)^a^
Sought medical care for last forced sex incident^f^	*N = 121*	*N = 40*	*N = 62*	*N = 41*
Yes	38.8	(40.2)^a^	34.4	(51.9)^a^
Used all services she needed^g^	*N = 393*	*N = 272*	*N = 391*	*N = 428*
Yes	19.4	32.7	40.9	25.7

^a^Bootstrap analysis was not possible because of too few observations in some categories. A weighted proportion was calculated instead

^b^N: Excludes FSWs who reported they wanted to get pregnant, were pregnant or were infertile

^c^N: Currently using contraception = Yes

^d^N: Unwanted pregnancy in the last five years = Yes

^e^N: Age > =30 years

^f^N: Forced sex in the past 12 months = Yes

^g^N: Was in need of at least one SRH service

**Table 3:** Pairwise comparison of SRH risks and use of SRH commodities and services across cities^a^

	Tete vs Durban		Mombasa vs Durban		Mysore vs Durban		Mombasa vs Tete		Mysore vs Tete		Mysore vs Mombasa	
	OR^b^	*p*-value	OR	*p*-value	OR	*p*-value	OR	*p*-value	OR	*p*-value	OR	*p*-value
Currently using contraception^c^												
Yes	0.76	0.988	6.74	0.001	3.96	0.979	8.81	0.001	5.17	0.942	0.59	1.000
Main contraception method used^d^												
Injectable contraceptives	1.94	0.110	0.91	0.999	<0.01	<0.001	0.47	0.016	<0.01	<0.001	<0.01	<0.001
Oral contraceptives	21.61	<0.001	6.50	0.106	0.51	0.979	0.30	0.002	0.02	<0.001	0.08	0.100
IUD	6.49	0.651	7.37	0.577	8.67	0.816	1.14	1.000	1.34	1.000	1.78	1.000
Implant	-	-	-	-	-	-	17.18	<0.001	0.34	0.538	0.02	<0.001
Condom	0.12	<0.001	0.29	<0.001	0.07	0.072	2.32	0.021	0.54	0.994	0.23	0.691
Female sterilization	0.19	0.377	0.08	0.150	79.36	0.004	0.42	0.988	411.6	0.001	988.9	<0.001
Currently using a non-barrier modern contraceptive method^c^												
Yes	4.11	<0.001	3.74	<0.001	6.98	0.059	0.91	0.999	1.70	0.982	1.86	0.958
Ever used emergency contraception												
Yes	0.62	0.598	1.76	0.062	0.11	<0.001	2.82	0.003	0.169	0.003	0.06	<0.001
Unwanted pregnancy in the last five years												
Yes	0.16	<0.001	0.86	0.985	0.18	<0.001	5.39	0.001	1.14	1.000	0.211	<0.001
Action taken for unwanted pregnancy^e^												
Went to a health facility	3.46	0.622	5.44	0.016	47.97	0.002	1.57	0.997	13.86	0.242	8.81	0.246
Ever tested for cervical cancer												
Yes	-	-	0.39	0.008	0.25	<0.001	-	-	-	-	0.63	0.673
Ever tested for cervical cancer^f^												
Yes	-	-	0.36	0.091	0.21	0.002	-	-	-	-	0.57	0.641
Forced sex in the past 12 months												
Yes	0.38	0.003	0.31	<0.001	0.21	<0.001	0.83	0.989	0.55	0.441	0.66	0.822
Condom use at last forced sex incident^g^												
Yes	0.43	0.841	0.31	0.168	2.68	0.599	0.72	0.998	6.21	0.275	8.64	0.040
Sought medical care for last forced sex incident^g^												
Yes	0.81	0.999	1.63	0.972	3.41	0.477	2.02	0.894	4.23	0.355	2.09	0.752
Used all services she needed^g^												
Yes	2.77	0.002	3.99	<0.001	1.76	0.622	1.44	0.498	0.64	0.872	0.44	0.287

^a^ Post-hoc pairwise comparison tests after fitting a logistic regression model with RDS-adjusted weights and adjusting for the confounding effect of individual sex worker characteristics

^b^Odds Ratio

^c^N: Excluding reason for not using contraception=’Want to get pregnant’, ‘Is currently pregnant’ or ‘Is unable to conceive’

^d^N: Currently using contraception = Yes

^e^N: Unwanted pregnancy in the last five years = Yes

^f^N: Age > =30 years

^g^N: Forced sex in the past 12 months = Yes

^h^N: Was in need of at least one SRH service

(Should appear in the chapter ‘Occurrence of SRH risks and use of SRH commodities and services (Table 2 and 3)’)

The majority of FSWs with a need for contraception (not wanting to become pregnant, not pregnant and not infertile) were using a contraceptive method in all cities, though the proportion was significantly higher in Mombasa than in Durban and Tete (*p* < 0.05). In all cities, but particularly in Durban and to a lesser extent Mombasa, a large proportion used condoms alone as contraceptive method. The proportion of FSWs who used a non-barrier contraceptive method was substantially lower in Durban (33%) than in Mombasa (66%), Tete (70%) and Mysore (85%) (*p* < 0.01). Of the non-barrier methods, injectable hormonal contraceptives were the most often used method in Durban and female sterilisation in Mysore. In Mombasa the implant was often used. Oral hormonal contraceptives were only commonly used in Tete and the IUD was rarely used across cities. Emergency contraception was less commonly used in Mysore (2.4% ever used it) than in the African cities (ranging from 13% in Tete to 38% in Mombasa) (*p* < 0.01).

In Durban and Mombasa about a third of FSWs reported to have experienced a pregnancy in the last 5 years, when they didn’t want to get pregnant at that time. This proportion was substantially lower in Tete and Mysore (7.6% and 8.0%, respectively, *p* < 0.01). In Durban, Tete and Mombasa, most FSWs kept the pregnancy, despite being unwanted, while in Mysore only a small proportion (6.3%) did so.

Cervical cancer screening was more commonly accessed in FSWs in Durban (29% reported to have ever been screened) than in the other cities (*p* < 0.01). If we only consider FSWs of at least 30 years old, the proportion of FSWs ever screened was higher, but still low in Mombasa and Mysore. In Tete, none of the interviewed FSWs had ever been screened for cervical cancer.

The proportion of FSWs who reported that in the past 12 months they were forced to have sex was highest in Durban (36%) and the lowest in Mysore (7.1%). Condom use during forced sex was reported by about half of the victims in Durban, but fewer in Tete and Mombasa. In all cities, a large proportion of the victims did not seek medical care following sexual violence.

Using the composite index, a similar proportion of FSWs (ranging between 25.8% and 32.7%) accessed all the services they needed in Durban, Tete and Mysore, but in Mombasa the proportion was significantly higher (40.9%) (*p* = <0.001, 0.058 and 0.006 compared to Durban, Tete and Mysore, respectively).

## Discussion

Our analysis shows that, prior to the implementation of FSW-targeted interventions by the DIFFER project, in all four cities, the use of certain SRH commodities and services, other than HIV prevention and care, was low. This is best summarised by our composite index of SRH use, which indicates that in only 20–40% of FSWs across the four cities, all SRH service needs were met. We also observed that several indicators of SRH care seeking behaviour differed greatly between the four cities. After adjusting for the – sometimes large - differences in socio-demographic and sex work characteristics of FSWs across cities, the differences in care seeking persisted and we therefore conclude that they are not likely to be due to differences in these measured characteristics. From the other components of the situational analysis, namely the focus group discussions, the key informant interviews and the health facility assessments, we learned that the availability and accessibility of SRH services differed substantially between cities and therefore believe that these structural and contextual factors play a more important role [24].

Excluding those women who either wanted to become pregnant or who reported that they were not able to conceive, self-reported contraception use was high, compared to many other surveys in similar settings [7–10, 30, 31] and to the prevalence of unmet need for family planning amongst married/cohabiting women of the general population [32–35]. However, a very large proportion of FSWs in Durban, and to a lesser extent in Mombasa, rely on condoms alone for contraception. Although condoms are considered an effective contraception method if consistently and correctly used [36], the use of condoms alone for contraception among FSWs has been shown to be ineffective in several studies, in particular because of inconsistent condom use with sexual partners other than clients [7–10]. The promotion of dual method use, consistent and correct condom use combined with a non-barrier contraceptive method, needs to be intensified in all cities, but particularly in Durban and Mombasa.

The absolute number of women who reported an unwanted pregnancy was often small and quantifying care seeking for an abortion was therefore difficult. Nevertheless, in all African cities a large proportion reported not to have gone to a health facility for an abortion and either kept the pregnancy or went somewhere else for an abortion, while in Mysore most went to a health facility. This is not surprising in Mozambique and Kenya, where termination of pregnancy (TOP) was illegal. In South Africa however TOP was legal and offered both at government and non-government facilities [37, 38] and it is surprising that only a small proportion went for an abortion. It is possible that some of these pregnancies were unplanned but not really unwanted, but that doesn’t explain why the use of TOP was so much lower in Durban than in Mysore. The reasons for FSWs not accessing TOP services in Durban needs to be further explored.

The few studies that have investigated cervical cancer screening in FSWs in resource-limited countries have shown a low uptake [39, 40], similar to the results shown in our study. Cervical cancer screening services do not appear to be sufficiently reaching FSWs, even those who are above 30 years. In Tete, none of the FSWs had to their knowledge ever been screened, despite the fact that screening is available at major health centres. In South Africa and Kenya, cervical cancer screening is not only part of routine sexual and reproductive care for women aged 30 years and older, but also of HIV care among women of all ages. We assessed if this was a possible explanation for the higher proportion of FSWs ever screened in Durban. The proportion of FSWs reporting to ever have been screened among those who reported to be on HIV care (ART or pre-ART) was indeed higher than among those not on HIV care (42.2% vs. 25.9%), but even among the women not enrolled in HIV care, the proportion was still substantially higher than in the other cities. Given that FSWs have multiple partners and are at higher risk of HPV infection than general population [12, 13], cervical cancer screening in these populations should be intensified in all cities, and particularly in Tete, Mombasa and Mysore.

As has been repeatedly shown in other studies in similar settings, violence against FSWs, including sexual violence, is common [14, 41–43]. The proportion of FSWs who reported to have been forced to have sex in the past year was particularly alarming in Durban and many reported to have been forced multiple times (data not shown). Also in the other cities forced sex appears not uncommon, and overall, half or more of those assaulted did not seek medical care for it. This clearly indicates a need for improving access to, and use of, such services in all cities. In addition, medical services for victims of forced sex need to be integrated within a comprehensive package of prevention and care services, including appropriate referral for social and legal support.

There are certain limitations inherent to our study design that have to be taken into account in the interpretation of the results. For RDS to be successful, the refusal rate needs to be low or at least similar among different type of FSWs. In Tete, refusal was common among Mozambican FSWs. After investigation by the peer educators, it appeared that many FSWs distrusted the confidentiality and intentions of the survey. We therefore believe that FSWs of Zimbabwean origin are over-represented. Further, responses given in interviewer administered face-to-face interviews might be influenced by poor understanding of the question, recollection bias, social desirability bias, or reluctance to divulge sensitive personal information [44]. We phrased the questions in the same way and used the same response options to minimise differential bias across cities, but can nevertheless not exclude that reporting bias could differ because of translations and different socio-cultural contexts. Lastly, we only assessed access to biomedical interventions that address the SRH of sex workers and not other important strategies such as legal support for those who had experienced violence.

## Conclusion

Female sex workers’ use of SRH commodities and services is often low and highly context-specific. The variations noted between cities are unlikely to be caused by differences in sex worker sociodemographic characteristics and more probably a result of differences in the availability of, and barriers to, SRH services. Access to services and uptake need to be improved in all cities. Intervention packages to improve utilization of contraceptive methods and services for cervical cancer screening, sexual violence (medical, social and legal) and termination of pregnancy should be tailored to the particular gaps in each city. The findings of the cross-sectional survey were triangulated with the findings of the other situational analysis components and informed the development of appropriate intervention packages.

## Abbreviations

ART: Anti-retroviral therapy
DIFFER: Diagonal interventions to fast-forward enhanced reproductive health
FSW: Female sex worker
HIV: Human immunodeficiency virus
HPV: Human papilloma virus
IUD: Intra-uterine device
RDS: Respondent driven sampling
SGBV: Sexual and gender-based violence
SRH: Sexual and reproductive health
TOP: Termination of pregnancy

## References

[1] Scorgie F, Chersich MF, Ntaganira I, Gerbase A, Lule F, Lo YR (2012). Socio-demographic characteristics and behavioral risk factors of female sex workers in Sub-Saharan Africa: a systematic review. Aids Behavior.

[2] Scorgie F, Vasey K, Harper E, Richter M, Nare P, Maseko S (2013). Human rights abuses and collective resilience among sex workers in four African countries: a qualitative study. Glob Health.

[3] Scorgie F, Nakato D, Harper E, Richter M, Maseko S, Nare P (2013). ‘We are despised in the hospitals’: sex workers’ experiences of accessing health care in four African countries. Culture Health Sex.

[4] Vuylsteke B, Ghys PD, Mah-bi G, Konan Y, Traore M, Wiktor SZ (2001). Where do sex workers go for health care? A community based study in Abidjan, Cote d’Ivoire. Sex Transm Infect.

[5] Delvaux T, Crabbe F, Seng S, Laga M (2003). The need for family planning and safe abortion services among women sex workers seeking STI care in Cambodia. Reprod Health Matters.

[6] Dhana A, Luchters S, Moore L, Lafort Y, Roy A, Scorgie F (2014). Systematic review of facility-based sexual and reproductive health services for female sex workers in Africa. Glob Health.

[7] Morineau G, Neilsen G, Heng S, Phimpachan C, Mustikawati DE (2011). Falling through the cracks: contraceptive needs of female sex workers in Cambodia and Laos. Contraception.

[8] Schwartz S, Papworth E, Thiam-Niangoin M, Abo K, Drame F, Diouf D (2015). An Urgent Need for Integration of Family Planning Services Into HIV Care: The High Burden of Unplanned Pregnancy, Termination of Pregnancy, and Limited Contraception Use Among Female Sex Workers in Cote d'Ivoire. Jaids-J Acquir Immune Defic Syndr.

[9] Sutherland EG, Alaii J, Tsui S, Luchters S, Okal J, King'ola N (2011). Contraceptive needs of female sex workers in Kenya -- A cross-sectional study. Eur J Contracept Reprod Health Care.

[10] Yam EA, Mnisi Z, Mabuza X, Kennedy C, Kerrigan D, Tsui A (2013). Use of Dual Protection Among Female Sex Workers In Swaziland. Int Perspect Sex Reprod Health.

[11] Feldblum PJ, Nasution MD, Hoke TH, Van Damme K, Turner AN, Gmach R (2007). Pregnancy among sex workers participating in a condom intervention trial highlights the need for dual protection. Contraception.

[12] Cwikel JG, Lazer T, Press F, Lazer S (2008). Sexually transmissible infections among female sex workers: an international review with an emphasis on hard-to-access populations. Sex Health.

[13] Soohoo M, Blas M, Byraiah G, Carcamo C, Brown B (2013). Cervical HPV infection in female sex workers: a global perspective. Open AIDS J.

[14] Decker MR, Crago AL, Chu SKH, Sherman SG, Seshu MS, Buthelezi K (2015). Human rights violations against sex workers: burden and effect on HIV. Lancet.

[15] Stacey M, Konstant T, Rangasami J, Stewart M, Mans G. Estimating the size of the sex worker population in South Africa. South African National AIDS Council, 2013.

[16] Lafort Y, Geelhoed D, Cumba L, Lazaro CDM, Delva W, Luchters S (2010). Reproductive health services for populations at high risk of HIV: Performance of a night clinic in Tete province, Mozambique. BMC Health Serv Res.

[17] Odek WO, Githuka GN, Avery L, Njoroge PK, Kasonde L, Gorgens M (2014). Estimating the Size of the Female Sex Worker Population in Kenya to Inform HIV Prevention Programming. Plos ONE.

[18] Luchters S, Chersich MF, Rinyiru A, Barasa MS, King'ola N, Mandaliya K (2008). Impact of five years of peer-mediated interventions on sexual behavior and sexually transmitted infections among female sex workers in Mombasa, Kenya. BMC Public Health.

[19] O'Brien N, Reza-Paul S, Akram P, Jai S, Venukumarc KT, Venugopalc MS (2011). Community-led structural invention's promise for HIV prevention: A case study from the Ashodaya Samithi sex worker collective of Mysore, India. Sex Transm Infect.

[20] ICRH. The DIFFER Project. http://differproject.eu/. Accessed 22 Dec 2016.

[21] Grodos D, Mercenier P. Health systems research: a clearer methodology for more effective action. In: ITGPress, editor. 2000.

[22] Lafort Y, Greener R, Roy A, Greener L, Ombidi W, Lessitala F (2016). HIV prevention and care-seeking behaviour among female sex workers in four cities in India, Kenya, Mozambique and South Africa. Trop Med Int Health.

[23] Lafort Y, Greener R, Roy A, Greener L, Ombidi W, Lessitala F (2016). Where do female sex workers seek HIV and reproductive health care and what motivates these choices? A survey in 4 cities in India, Kenya, Mozambique and South Africa. Plos ONE.

[24] The DIFFER Consortium. Report of situational analysis of reproductive health services for general population women and female sex workers in India, Kenya, Mozambique and South Africa. 2013. http://differproject.eu/Project_Documents. Accessed 22 Dec 2016.

[25] Lafort Y, Jocitala O, Candrinho B, Greener L, Beksinska M, Smit JA (2016). Are HIV and reproductive health services adapted to the needs of female sex workers? Results of a policy and situational analysis in Tete, Mozambique. BMC Health Serv Res.

[26] Lafort Y, Lessitala F, Candrinho B, Greener L, Greener R, Beksinska M (2016). Barriers to HIV and sexual and reproductive health care for female sex workers in Tete, Mozambique: results from a cross-sectional survey and focus group discussions. BMC Public Health.

[27] Gile KJ, Handcock MS. Respondent-driven sampling: an assessment of current methodology. In: Liao TF, editor. Sociological Methodology, Vol 40. Sociological Methodology; 2010. p. 285-327. 10.1111/j.1467-9531.2010.01223.x PMC3437336 22969167

[28] Salganik MJ, Heckathorn DD (2004). Sampling and estimation in hidden populations using respondent-driven sampling. Sociol Methodol 2004 Vol 34.

[29] Dinno A (2015). Nonparametric pairwise multiple comparisons in independent groups using Dunn's test. Stata J.

[30] Khan MR, Turner AN, Pettifor A, van Damme K, Rabenja NL, Ravelomanana N (2009). Unmet need for contraception among sex workers in Madagascar. Contraception.

[31] Wayal S, Cowan F, Warner P, Copas A, Mabey D, Shahmanesh M (2011). Contraceptive practices, sexual and reproductive health needs of HIV-positive and negative female sex workers in Goa, India. Sex Transm Infect.

[32] Department of Health MRC, OrcMacro (2007). South Africa Demographic and Health Survey 2003.

[33] Kenya National Bureau of Statistics. Kenya Demographic and Health Survey 2014. Nairobi, Kenya: Kenya National Bureau of Statistics; Ministry of Health; the National AIDS Control Council (NACC); the National Council for Population and Development (NCPD); the Kenya Medical Research Institute (KEMRI), 2015.

[34] International Institute for Population; Sciences (IIPS) and Macro International. National Family Health Survey (NFHS-3), 2005–06: India: Volume I. Mumbai, India: International Institute for Population; Sciences (IIPS) and Macro International, 2007.

[35] Instituto Nacional de Saúde (INS), Instituto Nacional de Estatística (INE), e ICF Macro (2013). Moçambique - Inquérito Demográfico e de Saúde 2011.

[36] WHO. Family planning: a global handbook for providers-2011 Update Johns Hopkins Bloomberg School of Public Health/Center for Communication Programs and World Health Organization; 2011.

[37] Mhlanga RE (2003). Abortion: Developments and impact in South Africa. Br Med Bull.

[38] Varkey SJ (2000). Abortion services in South Africa: available yet not accessible to all. Int Fam Plan Perspect.

[39] Hong Y, Zhang C, Li XM, Lin DH, Liu YJ (2013). HPV and cervical cancer related knowledge, awareness and testing behaviors in a community sample of female sex workers in China. BMC Public Health.

[40] Kietpeerakool C, Phianmongkhol Y, Jitvatcharanun K, Siriratwatakul U, Srisomboon J (2009). Knowledge, awareness, and attitudes of female sex workers toward HPV infection, cervical cancer, and cervical smears in Thailand. Int J Gynecol Obstet.

[41] Alemayehu M, Yohannes G, Damte A, Fantahun A, Gebrekirstos K, Tsegay R (2015). Prevalence and predictors of sexual violence among commercial sex workers in Northern Ethiopia. Reprod Health.

[42] Olufunmilayo IF, Abosede TD (2014). Prevalence and correlates of violence against female sex workers in Abuja, Nigeria. Afr Health Sci.

[43] Schwitters A, Swaminathan M, Serwadda D, Muyonga M, Shiraishi RW, Benech I (2015). Prevalence of rape and client-initiated gender-based violence among female sex workers: Kampala, Uganda, 2012. Aids Behavior.

[44] Langhaug LF, Sherr L, Cowan FM (2010). How to improve the validity of sexual behaviour reporting: systematic review of questionnaire delivery modes in developing countries. Trop Med Int Health.

